# Relative Age Effect on Motor Competence in Children Aged 4–5 Years

**DOI:** 10.3390/children8020115

**Published:** 2021-02-06

**Authors:** Rubén Navarro-Patón, Marcos Mecías-Calvo, José Eugenio Rodríguez Fernández, Víctor Arufe-Giráldez

**Affiliations:** 1 Facultad de Formación del Profesorado, Universidade de Santiago de Compostela, 27001 Lugo, Spain; ruben.navarro.paton@usc.es; 2 Facultad de Ciencias de la Salud, Universidad Europea del Atlántico, 39011 Santander, Spain; 3 Centro de Investigación y Tecnología Industrial de Cantabria (CITICAN), 39011 Santander, Spain; 4 Facultad de Ciencias de la Educación, Universidade de Santiago de Compostela, 15782 Santiago de Compostela, Spain; geno.rodriguez@usc.es; 5 Facultad de Ciencicias de la Educación, Universidad de A Coruña, 15008 A Coruña, Spain; varufe@udc.es

**Keywords:** manual dexterity, aiming and catching, balance, Movement Assessment Battery for Children-2 (MABC-2), childhood, preschool children

## Abstract

The purpose of this study was to evaluate whether a Relative Age Effect (RAE) exists in motor competence of preschool children. The hypothesis was that motor competence, assessed by the Movement Assessment Battery for Children-2 (MABC-2), would be higher in preschool children born in the first quarter of the year compared to those who were born in the last quarter of the same year. A total of 360 preschool children were evaluated of whom 208 (57.8%) were boys and 152 (42.8%) were girls, with a mean age of 4.52 years old (± 0.50). The distribution of the participants was 172 children aged 4 years old and 188 aged 5 years old. The data showed a main effect on the age factor in the total score of aiming and catching (*p* < 0.001) and in the total test score (*p* < 0.001), in the quarter of birth factor in all the dimensions studied (i.e., total score of manual dexterity (*p* < 0.001); total score of aiming and catching (*p* = 0.001); total score of balance (*p* < 0.001); total test score (*p* < 0.001)) and in the interaction between both factors (i.e., total score of manual dexterity (*p* = 0.005); total score of aiming and catching (*p* = 0.002); total score of balance (*p* < 0.001); total test score (*p* < 0.001)). Age and quarter of birth produce a RAE in 4 and 5-year-old preschool children’s motor competence.

## 1. Introduction

In several fields, such as education, boys and girls are grouped by chronological age, whereby the purpose is fair equality of opportunities, ensuring a more adequate and uniform educational process for all [1]. The Spanish educational system is an example of this style of grouping, where entry deadlines to certain courses for children is January 1 and the school year begins in mid-September [2,3]. Therefore, we could find students with up to twelve months of chronological age difference in the same class [4], by which there will be age differences and, therefore, potential differences in maturity and experience among members of a class-group [5]. The chronological age difference between subjects of the same age group is known as relative age [6] and its consequences as a Relative Age Effect (RAE) [1]. RAE seems to exist, partially, due to biological maturation differences within members of the same cohort, although social or behavioral factors, among others, can be decisive [7,8].

Thus, preschool children whose relative age is younger than that of their schoolmates, have potential consequences such as poorer academic results [9,10,11], worse physical condition [12], less participation in school sports activities [13], a higher percentage of abandoning sports practices [14] and a lower probability of being chosen in detection processes [15], as well as entry to the first teams by overcoming the selection processes that take place within the clubs [1]. In physical education (PE), this effect gives an advantage to those born in the first months of a year, for the mere fact of having been born earlier, arguing that the longer the practice, the better results they will obtain [6,16]. In this sense, PE in schools is not kept out of these consequences, since a RAE has been found in the different physical tests without distinction by gender, as well as an influence of the RAE and the PE marks obtained by schoolchildren [6,17,18,19].

Evaluation and selection may be biased in PE classes because of age groupings and, therefore, cut-off dates also occur. Because of this, RAE may have important implications [19,20], reinforcing the competence of more mature and older individuals [13,21]. Thus, we must not forget that the development of motor competence in boys and girls is one of the objectives of PE [22,23], and the development of motor competence is considered a critical element within a quality PE class since it provides an inclusive, qualified and meaningful opportunity for all children [24]. For these reasons, motor competence has become one of the most important contents to work on in compulsory schooling [25,26], as included in the study plans designed by educational administrations [23], because the development of each child is of special importance to them since motor skills are essential for their integral development [27,28].

Children in early childhood learn primarily through movement, manipulation and control of objects [29]. Motor development begins through the learning of motor skills known as fundamental motor skills (FMS) (control skills of locomotives and objects; that is, running, jumping, catching, throwing...) [30]. These FMS are the basis of their future for both movement and physical activity in preschool children. If they are not developed properly, they can lead to a low perception of motor competence in children [31]. This low perception of competence, together with the low mastery of FMS, will cause preschool children to participate less and less in physical activities [32] and this low participation continues into adolescence and adulthood [33]. In addition, it is known that the practice of physical activity helps preschool children to develop different physical, social and emotional skills [34] while producing adequate bone growth and muscular and physiological development [35]. Therefore, taking into account the lack of sufficient physical activity associated with the low development of motor competence and the low self-perception of competence, together with the high caloric intake of society in general and of children in particular, could lead to the contribution of obesity [35].

The importance of researching RAE in schools is that it could reduce discrimination against students with lower motor skills, allowing the school to create equal opportunities for all children. However, to our knowledge, there is no study about the existence of RAE on the motor competence of preschool children aged from 4 to 5 years old. This is considered a fundamental stage for preschool children’s motor development since it is known that age and gender are the main predictors of motor competence [36]. 

In this sense, the following research questions were formulated: Are there differences in the motor competence of preschool children according to the trimester of birth? In what motor skills are there big differences between preschoolers? Are preschool children born in the first trimesters of the year the ones with more motor competence compared to those born in the last trimesters? Are there differences in motor competence between 4- and 5-year-olds?

For all the above, the purpose of this study was to research the existence of RAE in the motor competence of preschool children from Galicia (Spain), hypothesizing that the motor competence, measured by the Movement Assessment Battery for Children-2 (MABC-2) battery, would be higher in preschool children born in the first quarter of the year compared to those born in the last quarter of the same year.

## 2. Materials and Methods

### 2.1. Study Design

A non-experimental cross-sectional observational descriptive design was carried out [37]. The variables of MABC-2 battery were the dependent variables, comparing them according to the age and quarter of birth.

### 2.2. Participants

A non-probabilistic selection of the sample was carried out according to the subjects and the geographical proximity of six public education centers in Galicia (Spain). In these centers, Pre-School and Primary Education is taught with the same specific curriculum within which motor development is found, with the same hours (9:15 to 14:00) and access to the playground (30 min daily, like all the children in school) and 2 classes of 50 min per week of PE.

A total of 400 preschool children (4–5 years old) were invited to participate in the study. The inclusion criteria were the following: provide informed consent signed by their parents or legal guardians; not suffer from illness or difficulty (physical or mental) that prevents participation in the development of the MABC-2 tests; not have a final score below the 15th percentile after the test. Below this percentile, children may have motor competence problems, altering the results. To do this, the traffic light system offered in the MABC-2 evaluator’s manual has been followed (see 2.3. Measures). In this sense, 40 were previously excluded for not providing the informed consent signed by their parents or legal guardians. Of the 360 preschool children tested, 56 were excluded for presenting significant motor skill difficulties (once the battery test began) and 72 for being at risk of motor skill problems and requiring monitoring. In both cases, the preschool children were below the 15th percentile of the battery; thus, they did not meet the inclusion criteria. Lastly, the sample consisted of 232 preschool children.

All preschool children were classified into quarters based on their quarter of birth (quarter 1 (q1.: born from January to March); quarter 2 (q2.: born from April to June); quarter 3 (q3.: born from July to September) and quarter 4 (q4.: born from October to December)) and age group (4 and 5 years).

### 2.3. Measures

The MABC-2 battery [38], adapted to the Spanish context by Graupera and Ruíz (2012) [39] was used. It has been shown to be valid and reliable for identifying changes in motor competence over time in preschool children [38,39,40,41] with very high inter-rater reliability [42]. This battery comprises the following eight standardized tests in three specific skills: Manual dexterity, posting coins, threading beads (both scored as the time in seconds taken to complete) and drawing trail (scored by the number of errors the subjects make); aiming and catching, catching a bean bag and throwing a bean bag onto mat (both scored by the number of successful attempts); and balance, one-leg balance (scored as the time recorded), walking on raised heels and jumping on mats (both scored as the number of correct attempts registered) [38].

This tool provides direct and scalar scores for each test, scalar scores for the dimensions with equivalent percentiles and a total test score with its scalar and percentile equivalent score.

### 2.4. Procedures

The administration of the educational centers was contacted and explained the objective of the study, with the teachers of the different groups of preschool children later included in this explanation. Subsequently, a written document was sent to the parents and/or legal guardians, explaining the objective, purpose, design and procedure of the study (data recording, analysis techniques and their subsequent use), the declaration of confidentiality, the voluntary participation and the possibility of withdrawing the child from the study at any time they wished.

Once accepted by the parents and/or legal guardians of the preschool children, the necessary sociodemographic data (age, date of birth, sex) were recorded, and the MABC-2 battery was evaluated. 

For the evaluation of each test, the general rules of application of the MABC-2 battery of the manual were followed. Detailed information on the battery was provided to each evaluator, with experience in the application of standardized tests and in working with children, for their good knowledge and prior preparation of each of the tests. To avoid researcher biases, the evaluators were trained in the knowledge of each test, in the practice of the application and demonstration of each one of them, and in the mastery of the application procedures, as well as in the completion of the quantitative data of the booklet evaluator (qualitative data has not been evaluated).

To calculate the chronological age and therefore the choice of the age range for the application of the tests, the date of birth was subtracted from the date of application of the tests. For example, if a child was born on May 1, 2013, and was evaluated on January 10, 2019, his chronological age was 5 years, 6 months and 9 days.

To present the instructions for each test, the evaluators presented it in the same way: description of the task, demonstration by the examiner, practice by the child of the test as indicated in the procedure (where the examiner could correct possible errors) and execution of the test following the instructions of the manual (no instructions were given during the test performing).

Each child was individually assessed by the evaluators to increase the trust between the evaluator and the child and thus minimize the distractions of a group evaluation. The evaluation was carried out in an isolated classroom, bright, without obstacles, well ventilated and isolated from noise that could produce disturbances or interferences in the evaluation, which were provided by the educational centers. The room was provided with a table and two chairs for the tests that required sitting (i.e., manual dexterity).

After completing all the tests, for each of the eight tests, direct scores are obtained that are transformed into scalars following the ranges offered in the MABC-2 battery evaluator manual. From these 8 tests, the three dimensions of the MABC-2 (that is, manual dexterity, aiming and catching, and balance) and the total score are calculated from them. The scalar and percentile scores can be calculated from them, based on age, with the help of the manual. In addition, the total score can be interpreted in terms of a “traffic light” system designating three zones. i.e., green, performance within normal range (percentile above 16th); amber, performance within the “risk zone” where the child needs careful monitoring (6–15 percentile); red, motor competence problems (percentile less than 5).

For our study, the scalar measures of the three dimensions and the scalar and percentile scores of the total test score were used. It must be taken into account that in this battery, a high score in the different elements has a negative meaning of greater difficulty in its realization; therefore, the lower the score, the higher motor competence [43].

All research was carried out in accordance with the Declaration of Helsinki. Research protocol was sent to the Ethics Committee of the national Educa platform for review and its approval. The protocol was approved with the code number 22019.

### 2.5. Statistical Analysis

For the sociodemographic data analysis, the variables were expressed using frequency tables for categorical variables and central tendency measures for quantitative variables (mean and standard deviation). The differences in all the variables of the MABC-2 battery across the quarter of birth categories (q1. ys. q2. vs. q3. vs. q4.) and the age group (4 years old and 5 years old) were evaluated using a multivariate analysis of variance (MANOVA). The effect size was calculated using partial eta squared (η^2^), and the interaction between variables, using the Bonferroni statistic to learn of the significance. SPSS software (SPSS v.25, IBM Corporation, New York, USA) was used for all statistical analyses. The level of significance was set at *p* < 0.05. 

## 3. Results

A total of 232 healthy preschool children were evaluated of whom 116 (50.0%) were boys and 116 (50.0%) were girls (mean age of 4.51 years old (± 0.50)). The distribution of the participants was 112 preschool children aged 4 years old (*n* =28 (25%), *n* = 52 (46.4%), *n* = 20 (17.9%) and *n* = 12 (10.7%)), from quarter 1, quarter 2, quarter 3 and quarter 4, respectively, and 120 preschool children aged 5 years old (*n* =36 (30.0%), *n* = 44 (36.7%), *n* = 24 (20.0%) and *n* = 16 (13.3%)), from quarter 1, quarter 2, quarter 3 and quarter 4, respectively.The results of the MANOVAs (Table 1) regarding manual dexterity indicated that there is a significant main effect by the birthdate quarter factor (*F* (3, 232) = 246.87, *p* < 0.001, η^2^ = 0.06), which is higher in those born in the first quarter. A significant effect was found in the interaction between both factors (*F* (3, 232) = 4.403, *p* = 0.005, η^2^ = 0.23), but not in the age factor (*p* = 0.521). 

Regarding aiming and catching, the findings indicated that there is a significant main effect in the birthdate quarter factor (*F* (3, 232) = 5.362, *p* = 0.001, η^2^ = 0.07), with higher scores in those born in the second semester. In the age factor, a main effect was found (*F* (1, 232) = 17.651, *p* < 0.001, η^2^ = 0.07), with higher scores in 4-year-old preschool children. Statistical differences were found in the interaction between both factors (*F* (3, 232) = 4.929, *p* = 0.002, η^2^ = 0.06). 

Regarding balance, the results of the MANOVA indicated that there is a significant main effect of the birthdate quarter factor (*F* (3, 1415) = 10.791, *p* < 0.001, η^2^ = 0.13), with the scores higher in those born in the first quarter. Interaction effects were found between both factors (*F* (3, 232) = 6.956, *p* < 0.001, η^2^ = 0.08) but not in the age factor (*p* = 0.533). 

The results with respect to the total test score indicated that there is a significant main effect of the birthdate quarter factor (*F* (3, 232) = 16.854, *p* < 0.001, η^2^ = 0.18), with higher scores achieved by those born in the first quarter again. A main effect in the age factor was found (*F* (1, 232) = 14,159, *p* < 0.001, η^2^ = 0.06), with higher scores in 4-year-old preschool children. Likewise, there is also a main effect in the interaction between the birthdate quarter and age factors (*F* (3, 232) = 14.893, *p* < 0.001, η^2^ = 0.17).

Regarding the comparison by pairs, with respect to the manual dexterity, statistically, significant differences were found between the 4-year-old preschool children compared to the 5-year-olds in the first quarter (*p* = 0.022) and in the second quarter (*p* = 0.013), with higher scores in the youngest. Significant differences were found in the fourth quarter (*p* = 0.028), but the scores are now higher in the 5-year-old preschool children. Regarding the aiming and catching, statistically significant differences were found between 4-year-old and 5-year-old preschool children, being greater in the youngest, between those in the first (*p* < 0.001) and the third quarter (*p* < 0.001). When balance is analyzed, statistically significant differences were found between 4 and 5-year-old preschool children (*p* = 0.001), in favor of those with 4 years, also being higher in 5-year-olds in the fourth quarter (*p* = 0.003). Regarding the total test score, the results in the first three quarters appear higher scores in the 4-year-old preschool children (i.e., q1. (*p* < 0.001; q2. (*p* = 0.010); q3. (*p* = 0.004)), on the contrary when the fourth quarter (q4.; *p* = 0.001) is compared.

In the pairwise analysis based on age and the quarter of birth, with respect to the manual dexterity, differences were found in 4-year-old preschool children between q1. vs. q2. (*p* < 0.001); between q1. vs. q3. (*p* < 0.001) and between q1. vs. q4. (*p* < 0.001). The same occurs with the 5-year-old preschool children (i.e., q1. vs. q2. (*p* < 0.001); q1. vs. q3. (*p* = 0.008); q1. vs. q4. (*p* = 0.024)). In the aiming and catching in 4-year-old preschool children, only significant differences were found between those born in q1. vs. q3. (*p* = 0.025), between q2. vs. q3. (*p* = 0.015) and between those of q3. vs. q4. (*p* = 0.001). In 5-year-olds, there are significant differences between q1. vs. q2. (*p* < 0.001) and between q2. vs. q3. (*p* = 0.032). In the balance for 4-year-old preschool children, there are differences between q1. vs. q2. (*p* = 0.001), between q2. vs. q3. (*p* = 0.022) and between q1. vs. q4. (*p* < 0.001). In the total test score, significant differences were only found in 4-year-old preschool children between q1. vs. q2. (*p* < 0.001); q1. vs. q3. (*p* = 0.029); q1. vs. q4. (*p* < 0.001); q2. vs. q4. (*p* < 0.001) and q3. vs. q4. (*p* < 0.001).

**Figure 1:**
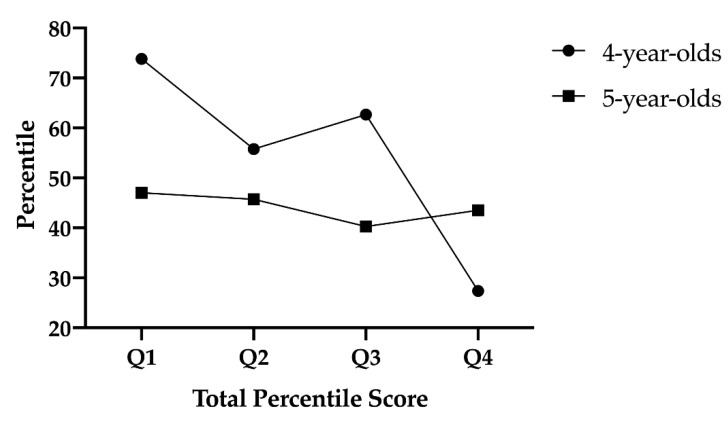
Percentiles according to age and quarter of birth. **Note:** Q: Quarter.

Although there are differences between the percentile reached by preschool children aged 4 and 5 years of age when the quarters are compared (Figure 1), the trend within each age group indicates that there are statistical differences between preschool children born in q1. vs. q2. (Mean = 73.85, SEM = 2.81 vs. Mean = 55.80, SEM = 3.08; (*p* < 0.001)); q1. vs. q3. [Mean = 73.85, SEM = 2.81 vs. Mean = 62.67, SEM = 3.0; (*p* = 0.049)] and finally q1. vs. q4. (Mean = 73.85, SEM = 2.81 vs. Mean = 27.40, SEM = 1.01; (*p* < 0.001)). The same occurs with 5-year-old preschool children but without statistical significance; q1. vs. q2. (Mean = 47.00, SEM = 2.22 vs. Mean = 45.72, SEM = 3.28; (*p* = 0.742)); q1. vs. q3. (Mean = 47.00, SEM = 2.22 vs. Mean = 40.25, SEM = 5.81; (*p* = 0.192)) and finally q1. vs. q4. (Mean = 47.00, SEM = 2.22 vs. Mean = 43.50, SEM = 3.30; (*p* = 0.440)).

## 4. Discussion

The purpose of this study was to evaluate RAE on motor competence of preschool children in Galicia, Spain. We hypothesized that motor competence, as measured by the MABC-2 battery, would be higher in preschool children who were born in the first quarter of the year compared to who were born in the last quarter of the same year.

The data obtained in our research indicates that motor competence improves as relative age increases in preschool children. The improvements, although with a tendency to increase as age increases within each group (4 and 5 years), are only significant among 4-year-old preschool children. In fact, a difference of 3 months in the date of birth in preschool children can represent up to 8% of their life [5]. These data should be taken into account by teachers educating for these ages, since a more short-term programming would be necessary to adapt to the specific physical development of each age.

The analyses carried out indicate the presence of RAE in each of the dimensions studied [i.e., manual dexterity; aiming and catching; balance and total test score]; the effect differs according to the age group from the interaction between the quarter of birth. RAE in school and sport seems to improve due to the fact that an individual will increase their performance simply because they were born earlier, maintaining that the more they practice, the better the results [6,16] or because this advantage of relatively older children is due to differences in physical size and biological maturation by the simple fact of growth [8,33], and this has not been the case in our study. Four-year-olds have the highest total score, contrary to expectations, that is, an increase in competence and motor development with age [8,44]. Four-year-old preschoolers have higher scores in manual dexterity, aiming and catching, and balance in the first three quarters of the year compared to 5-year-old preschool children, meaning that the total score for the smallest is superior to the older ones [45,46]. This does not follow the line proposed by Sánchez-González et al. (2011) [47], whose study indicates that although physical growth may be higher in 5-year-old school-age children [45], this factor may be a limiting factor for 4-year-old children. In addition to the previous study, there are others in which older preschool children have better average scores in terms of motor competence than younger ones [38] because they have better balance [46] or manual dexterity [48,49].

In manual dexterity and balance tests, the differences between the quarters of birthdate were significant. Those born in the first quarter obtained higher scores than those born in the second, third and fourth, as the relative age was higher (q1. > q2. > q3. > q4.), within each age group (4 and 5 years). This may be due to the fact that motor competence improves as children biologically mature and grow [50]. In fact, scientific evidence reports better results in older children when compared within their class-group [51,52,53]. The same happens as children get older, since, for example, balance improves to a great extent linearly between the ages of 2 and 18 [54] and manual dexterity usually develops during the preschool period [55].

Our results show linear changes based on relative age in the different dimensions of scores studied in 4-year-old preschool children, while it is not fulfilled in 5-year-olds [13]. The lack of progressive increase in motor competence could be due to the proximity of a critical period in our sample, biological maturity, the practice, or lack thereof, of physical activities and the heterogeneity on motor experiences that have not been taken into account in our research. In our research results, the only dimension that does not seem to follow this behavior at some point is aiming and catching tests [5]. In the aiming and catching, a reduction in the score obtained by older children was recorded. This twin interaction appears due to the few of differences between 5-year group quarters, and the different progressions observed, which in 4-year group scores is higher in quarter 3 preschool children than quarter 1 [56].

On the other hand, these analyses carried out showed main effects by age and quarter of birth. It means that motor competence improves as the age of the group advances [45,46,48,55,57]. In this way, it is considered that RAE is consistent across these different age groups, and that the same variable behaviors are expected in the performance of children born in different quarters within that age group [5,49]. Regarding the total percentile reached by preschool children, it increases from q4. to q1., so it is progressive quarter by quarter. Cases have been observed in which this percentile is equal between two consecutive quarters, as in the 5-year-olds age group (that is, q1. is similar to q2. and q3. is similar to q4.). Finally, as far as we know, this is the first study on RAE in preschool children, so we cannot compare the multivariable analysis results with previous studies.

RAE in sports selection and physical fitness were reported in large bodies of literature, but less on motor competence in preschool children (4–5 years). Therefore, the results of this study emphasize the fact that preschool children grouped in the same class year may show different motor competences within a period as short as one quarter (3 months). The results of this study should help to understand that the cut-off age is an important factor that influences the acquisition of skills in various areas (physical, cognitive, etc.) of child development [58,59]. The existence of RAE must be considered and compensated individually, according to the motor needs of preschool children, since younger students may reach the same level as their older peers in the future [56,60]. Therefore, the strategies for PE interventions and individual should be taken into account when teaching and evaluating children within the same academic year, starting at an early age such as preschool [8,61,62]. Strategies that could be implemented in schools to try to reduce RAE could be to group preschool children according to their biological and non-chronological age [63,64,65]; educating teachers about this RAE, so that they take this effect into account when carrying out assessments in PE classes [61,62,66] or applying corrective adjustments [67] to work on the contents for these ages in PE [68].

In this sense, the following practical recommendations are proposed: (1) Design and implement curricular interventions by teachers, for the specific work of motor competence through the creation of motor situations that present challenges and activities that allow children of age preschool develop their potential for movement throughout the academic year [69]. This should be done with the following premises: Tasks should be organized in such a way that they present a logical progression but with a certain level of challenge; individualize work by learning levels, adapting it to the student’s motor competence levels; and contribute to the success in the different tasks to motivate the student. (2) Combine more free play time and directed PE during the school day since the time of motor experiences is a determining factor in the development of motor competence [31]. (3) Implement programs based on FMS in other school settings, such as during recess and breaks in the classroom [28].

Regarding the limitations, the study sample may not be representative because the characteristics of the type of center and the geographical distribution were not random. On the other hand, we did not analyze sports attendance or anthropometric parameters of preschool children, factors to consider as they could explain RAE at these ages [7]. More studies are needed on this topic.

## Figures and Tables

**Table 1:** Results of Movement Assessment Battery for Children-2 (MABC-2) test based on age and the quarter of birthdate.

		Quarter 1		Quarter 2		Quarter 3		Quarter 4	
Total Scores	Years Old	Mean	SEM	Mean	SEM	Mean	SEM	Mean	SEM
Manual dexterity	4	34.00	0.62	29.85 *	0.41	29.33 *	0.37	28.00 *^,^**	0.45
	5	32.11 ^†^	0.28	28.18 *^,†^	0.77	29.50	0.74	30.16 ^†^	0.50
Aiming and catching	4	17.43	3.12	18.07	5.03	20.67 *	3.44	15.40 **^,^***	3.08
	5	13.55 ^†^	0.63	17.36 *	0.71	14.71 ^†^	1.02	15.66	0.69
Balance	4	36.00	0.69	33.00 *	0.37	33.00 *	3.90	28.40 *^,^**^,^***	3.97
	5	32.66 ^†^	0.59	32.27	0.64	32.25	1.14	31.83 ^†^	0.88
Total test Score	4	87.42	1.17	80.92 *	0.98	83.00 *	1.23	71.80 *^,^**^,^***	0.36
	5	78.33 ^†^	0.69	77.81 ^†^	1.13	76.50 ^†^	0.46	77.66 ^†^	1.04

**Note.** SEM: Standard Error of Mean; * *p* < 0.05 different to quarter 1; ** *p* < 0.05 different to quarter 2; *** *p* < 0.05 different to quarter 3; † *p* < 0.05 different to 4 years old.

## Data Availability

The data presented in this study are not available in accordance with Regulation (EU) of the European Parliament and of the Council 2016/679 of April 27, 2016 regarding the protection of natural persons with regard to the processing of personal data and the free circulation of these data (RGPD).
